# Longitudinal Neuroimaging of a Pediatric Patient With Sturge–Weber Syndrome: From Birth to Adolescence

**DOI:** 10.7759/cureus.95748

**Published:** 2025-10-30

**Authors:** Balwant Rai, Bhavisha Chaudhari, Eiman Abubaker, Saba Jaradat, Shalini Nandish

**Affiliations:** 1 Radiology, Calderdale and Huddersfield National Health Service (NHS) Foundation Trust, Huddersfield, GBR

**Keywords:** calverial hypertrophy, cerebral atrophy, choroidal hemangioma, cortical calcification, leptomeningeal angioma, pediatric brain mri, pediatric mri, port wine stain, sturge-weber syndrome

## Abstract

Sturge-Weber syndrome (SWS) is a rare congenital neurocutaneous disorder characterized by facial port-wine stains, leptomeningeal angiomas, and various ocular abnormalities. Serial neuroimaging provides critical insights into disease progression. We report a longitudinal case of a female patient with SWS followed from one month to 12 years of age. Imaging demonstrated leptomeningeal angiomas, progressive underlying brain atrophy, cortical calcifications, choroid plexus enlargement, and hemi-calvarial hypertrophy correlating with the patient’s clinical deterioration and seizure activity. This case highlights the predictable evolution of radiological features in SWS and underscores the importance of early MRI evaluation for confirming the diagnosis and long-term imaging follow-up for management, as well as prognostication.

## Introduction

Sturge-Weber syndrome (SWS) is a rare, sporadic, congenital neurocutaneous disorder that belongs to the group of phakomatoses. First described by Sturge in 1879 and later expanded upon by Weber in 1922, the syndrome is most often recognized by its characteristic clinical triad: a facial port-wine stain (nevus flammeus), seizures, and ocular abnormalities such as glaucoma or choroidal hemangiomas [1]. Unlike other phakomatoses, SWS is not inherited.

The condition has an estimated prevalence of approximately one in 20,000 to 50,000 live births [2], making it an uncommon but clinically significant disorder due to its diverse manifestations [3,4]. Neuroimaging plays a central role in both diagnosis and ongoing management, providing critical insights into the hallmark abnormalities of SWS. These include pial angiomatosis i.e abnormal proliferation of small blood vessels in the pia mater, cortical atrophy, progressive white matter changes, and parenchymal calcifications, which are best appreciated through imaging. Imaging (MRI and CT) is particularly valuable, as it not only aids in confirming the diagnosis but also in tracking disease progression, monitoring therapeutic responses, and anticipating neurological outcomes. 

In this report, we present the case of a female child with Sturge-Weber syndrome (SWS) who was followed from birth through adolescence. By highlighting the progressive radiological changes observed over time and their correlation with seizure burden, this case underscores the importance of serial neuroimaging in elucidating the natural history of SWS and guiding individualized patient management.

## Case presentation

**Figure 1:**
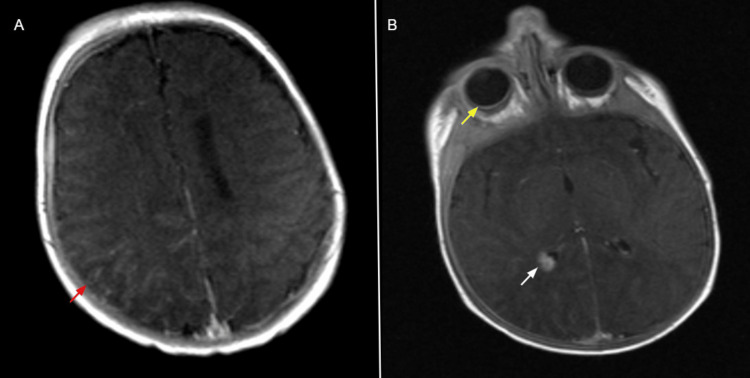
Contrast-enhanced MRI brain image at one month of age Post-contrast T1-weighted non-fat-saturated axial image (red arrow in A) demonstrates enhancing pial angiomas over the right cerebral hemisphere. Post-contrast T1-weighted non-fat-saturated axial image (yellow arrow in B) shows a diffuse enhancing choroidal hemangioma of the right eye and  (White arrow in B) indicates asymmetric enlargement of the right choroid plexus, confirming the diagnosis of Sturge–Weber syndrome.

A female child was born with a facial port-wine stain, raising early suspicion for Sturge-Weber Syndrome (SWS). At one month of age, contrast-enhanced MRI confirmed the diagnosis, demonstrating right cerebral pial angiomatosis, enlargement of the right choroid plexus, and a diffuse choroidal haemangioma involving the right eye (Figure 1).

**Figure 2:**
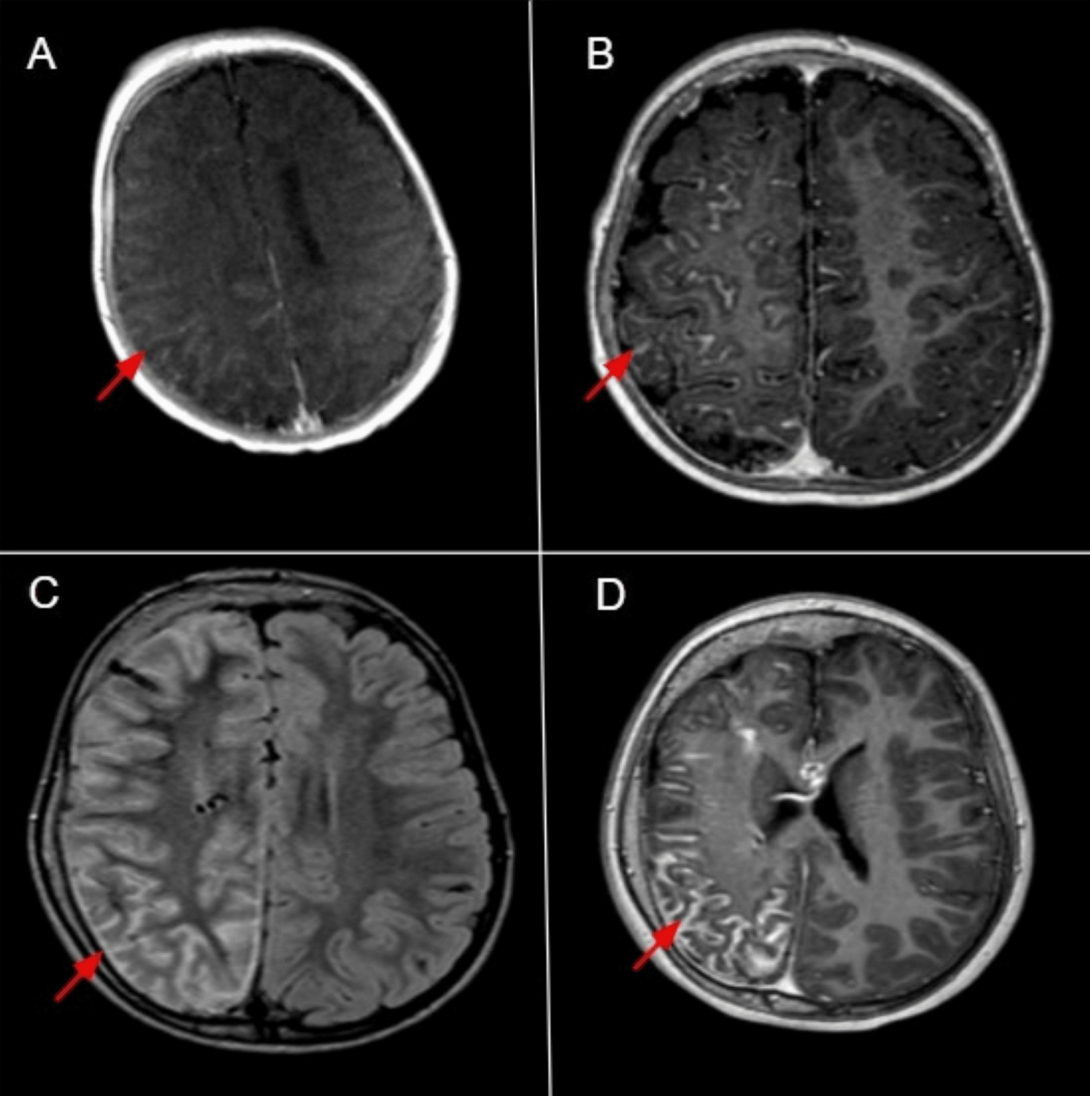
Results of imaging studies done at one month, one year, and 12 years of age Red arrows in A, B, and D (axial post-contrast T1-weighted non-fat-saturated images at one month, one year, and 12 years of age, respectively) demonstrate enhancing pial angiomatosis. The red arrow in C (axial non-contrast Fluid-Attenuated Inversion Recovery (FLAIR) image at six years of age) shows pial angiomatosis as sulcal hyperintensity with underlying parenchymal hyperintensity reflecting hypoxic injury. These images illustrate the persistent but increasingly prominent appearance of angiomatosis with age, largely due to progressive underlying cerebral atrophy.

Over time, she developed progressive neurological manifestations, most notably recurrent seizures and repeated episodes of left-sided weakness. Serial neuroimaging documented the characteristic evolution of SWS as detailed below. The pial angiomatosis persisted and became more pronounced with age, as shown in Figure 2.

**Figure 3:**
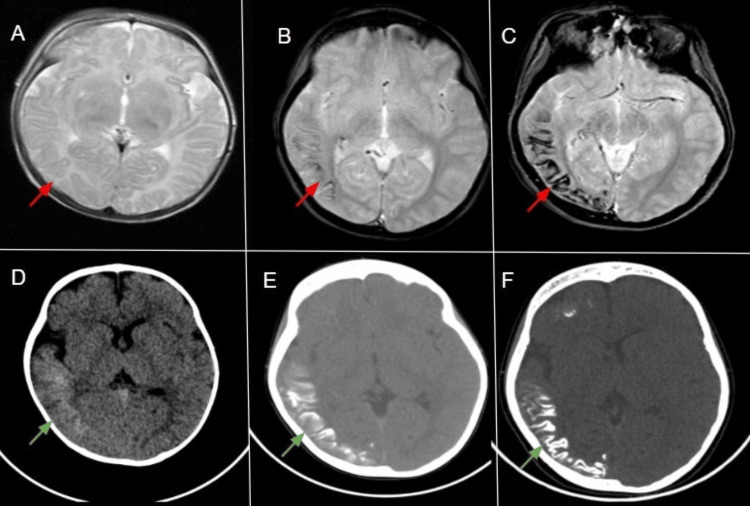
Axial GRE images Axial Gradient Echo (GRE) images (A–C) show progressive gyriform calcifications as blooming artifacts (red arrows) at one month, five years, and 12 years of age. Corresponding axial CT images (D–F) demonstrate the same process (green arrows) at one year, six years, and 10 years, illustrating the age-related progression of calcifications in Sturge–Weber syndrome.

Progressive gyriform calcifications developed within the affected cortex with age, as shown in Figure 3.

**Figure 4:**
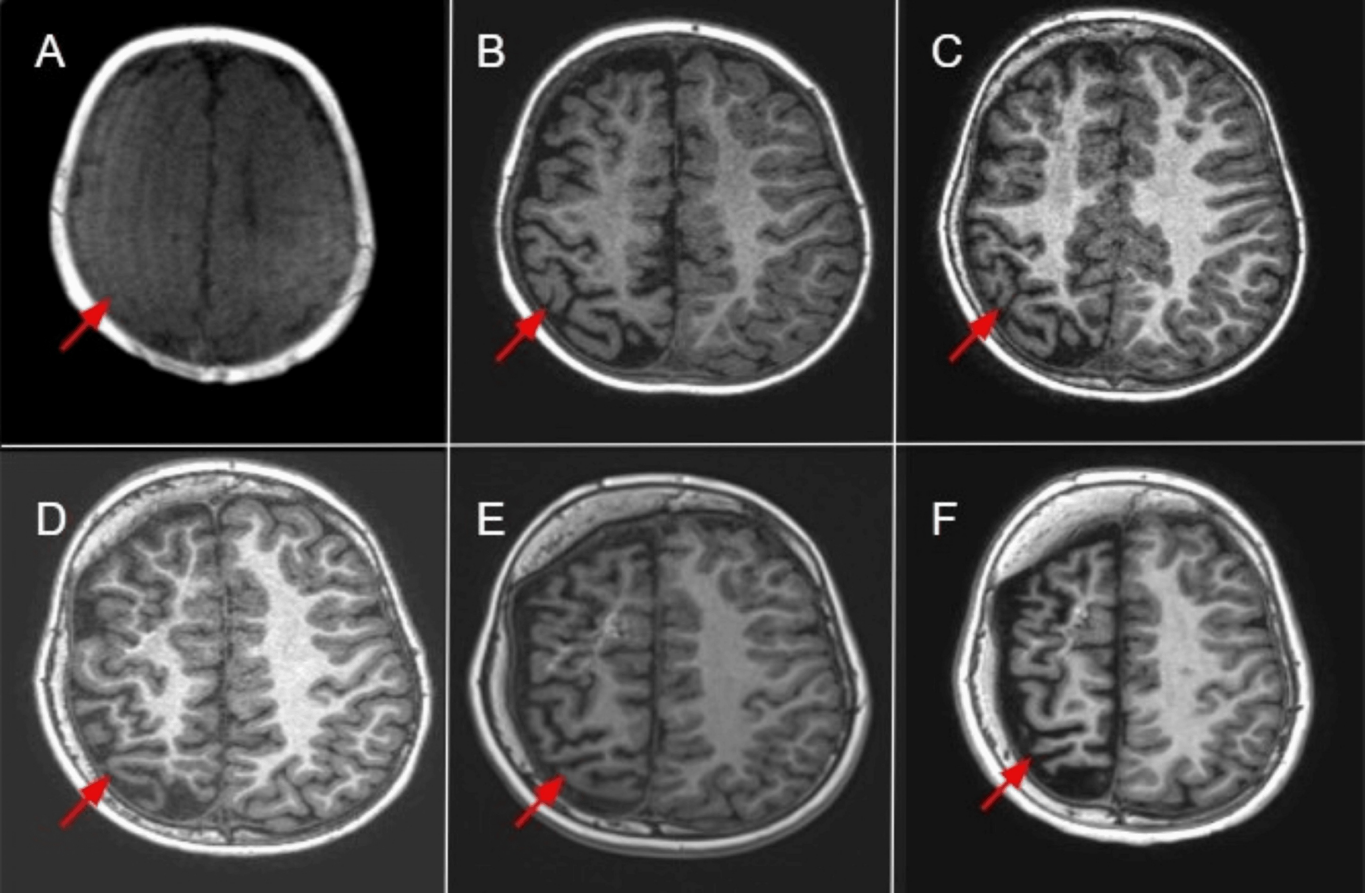
Axial non-fat-saturated T1-weighted images Axial non-fat-saturated T1-weighted images (A–F) demonstrate progressive right cerebral atrophy (red arrows) at one month, one year, five years, six years, 10 years, and 12 years of age, respectively.

Increasing atrophy of the right cerebral hemisphere was also noted with age, as shown in Figure 4.

**Figure 5:**
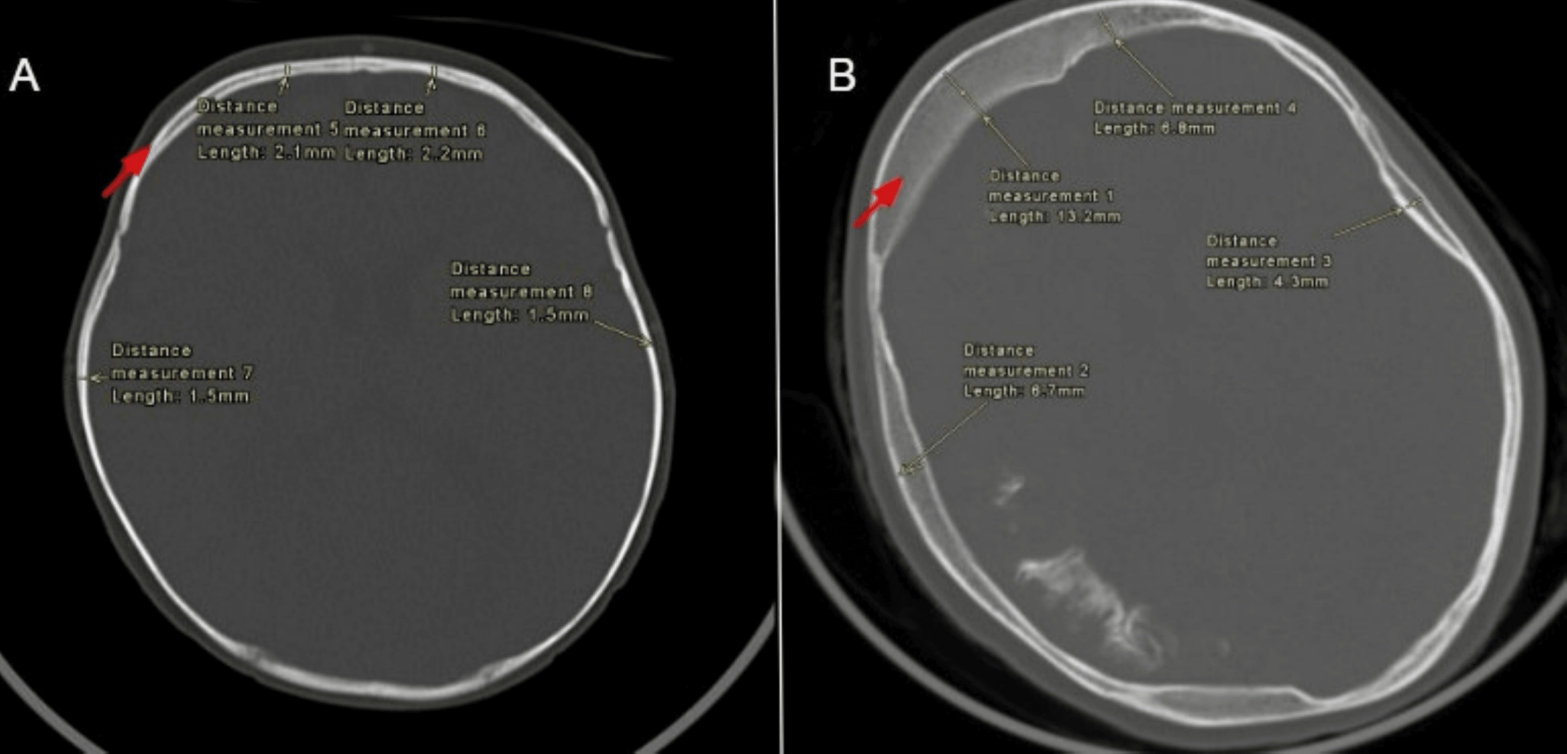
Axial CT bone window images at one year and 11 years of age Axial CT bone window images at one year (A) and 11 years (B) demonstrate progressive right-sided bony hemi-calvarial hypertrophy (red arrows), confirmed by interval increase in calvarial thickness measurements, i.e., from the initial 1-2 mm to follow-up thickness of 4-14 mm.

Secondary compensatory changes included hypertrophy of the ipsilateral hemi-calvarium, as shown in Figure 5.

**Figure 6:**
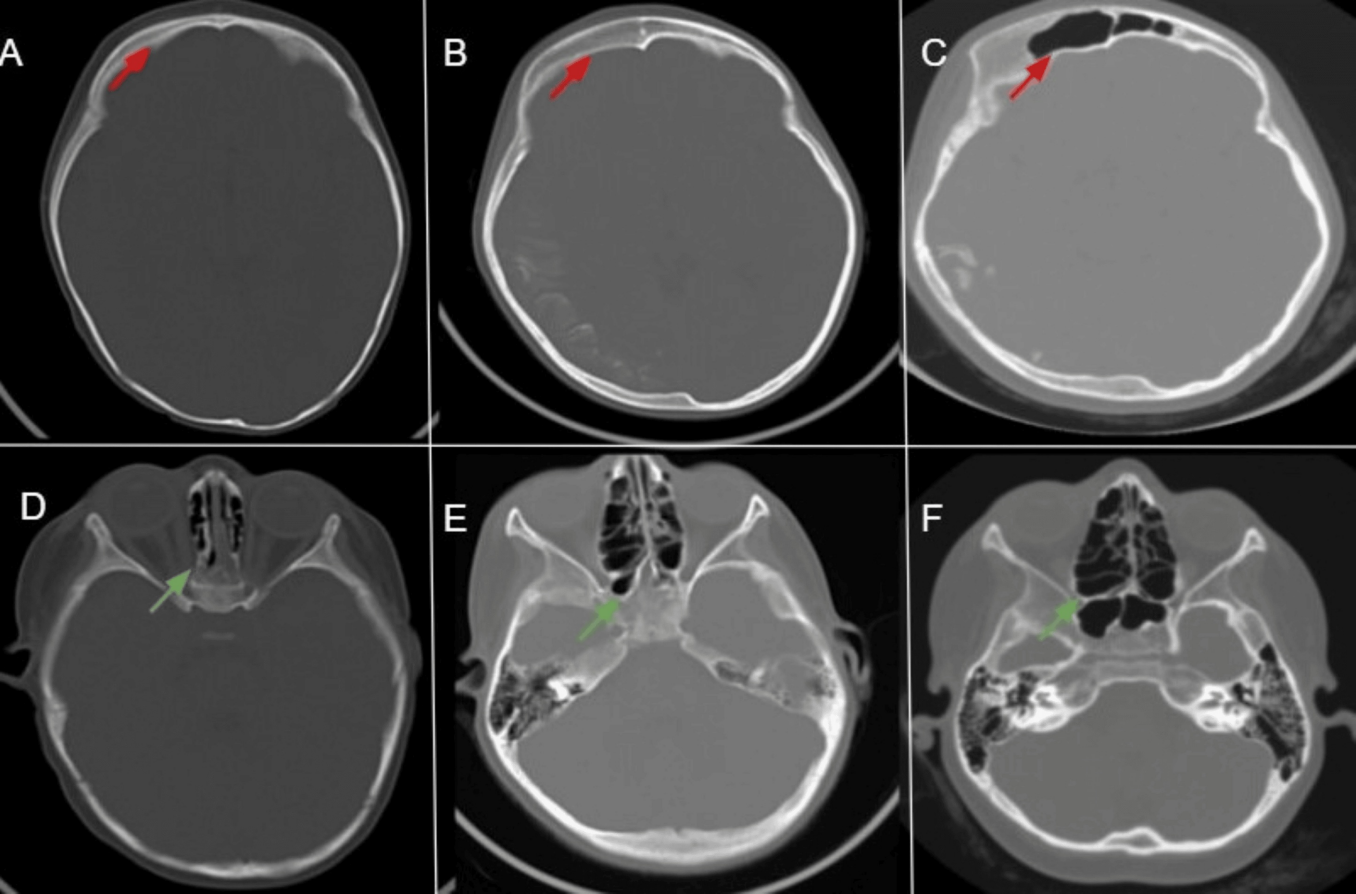
Axial CT bone window images Axial CT bone window images (A–C) demonstrate frontal sinus pneumatization with right-sided enlargement (red arrows) at one, six, and 11 years of age, respectively. Images (D–F) show ethmoidal and sphenoidal sinus pneumatization with right-sided enlargement (green arrows)at one, six, and 11 years of age, respectively.

Enlargement of the ipsilateral paranasal sinuses was also observed with age, as shown in Figure 6.

**Figure 7:**
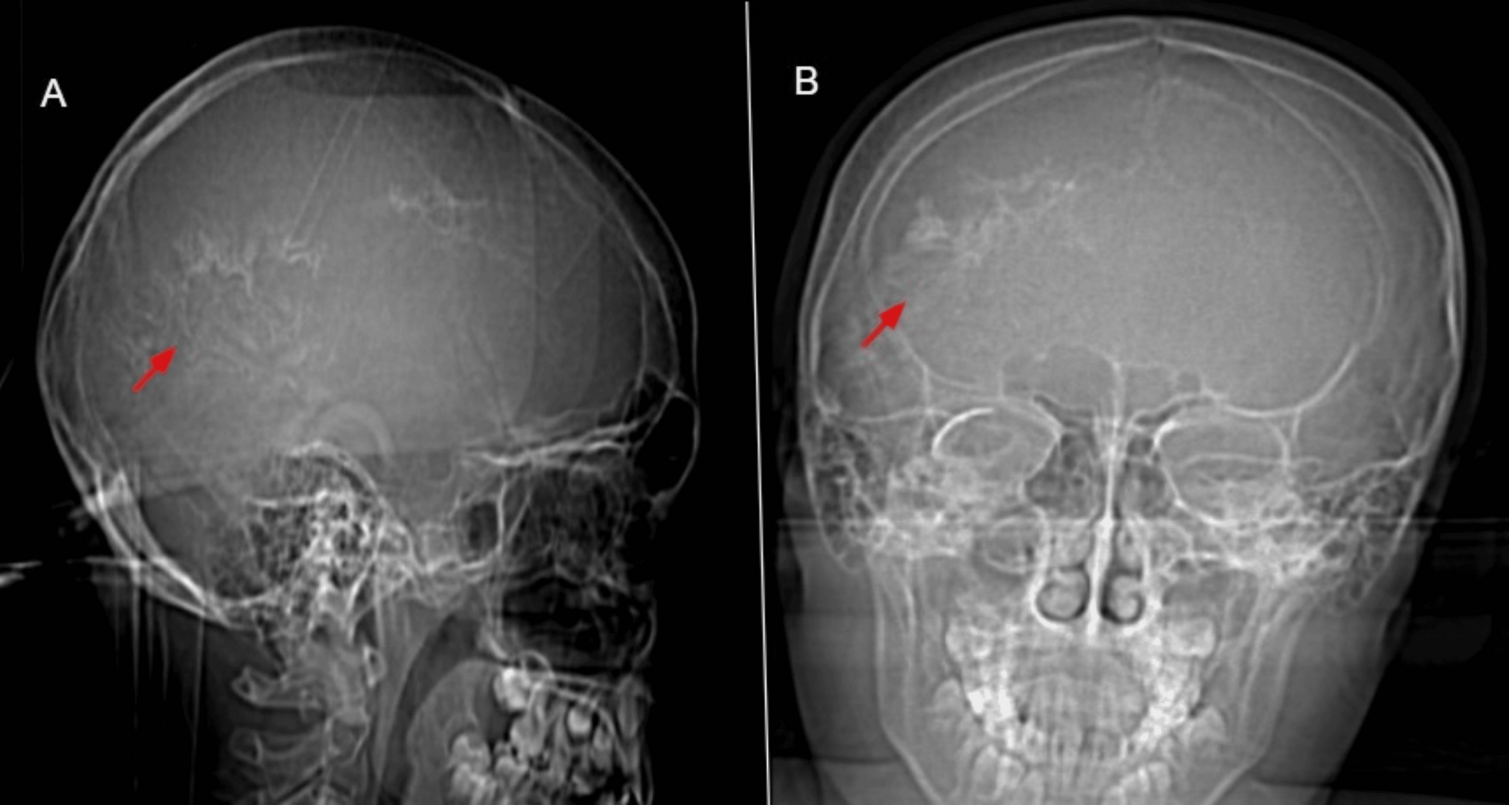
Lateral and frontal scout CT images at six years of age Lateral (A) and frontal (B) scout CT images at six years of age demonstrate right-sided gyriform ‘tram-track’ calcifications of the skull, corresponding to underlying parenchymal calcifications.

Scout CT images highlighted the classic “tram-track” pattern of gyriform calcifications, as shown in Figure 7.

**Figure 8:**
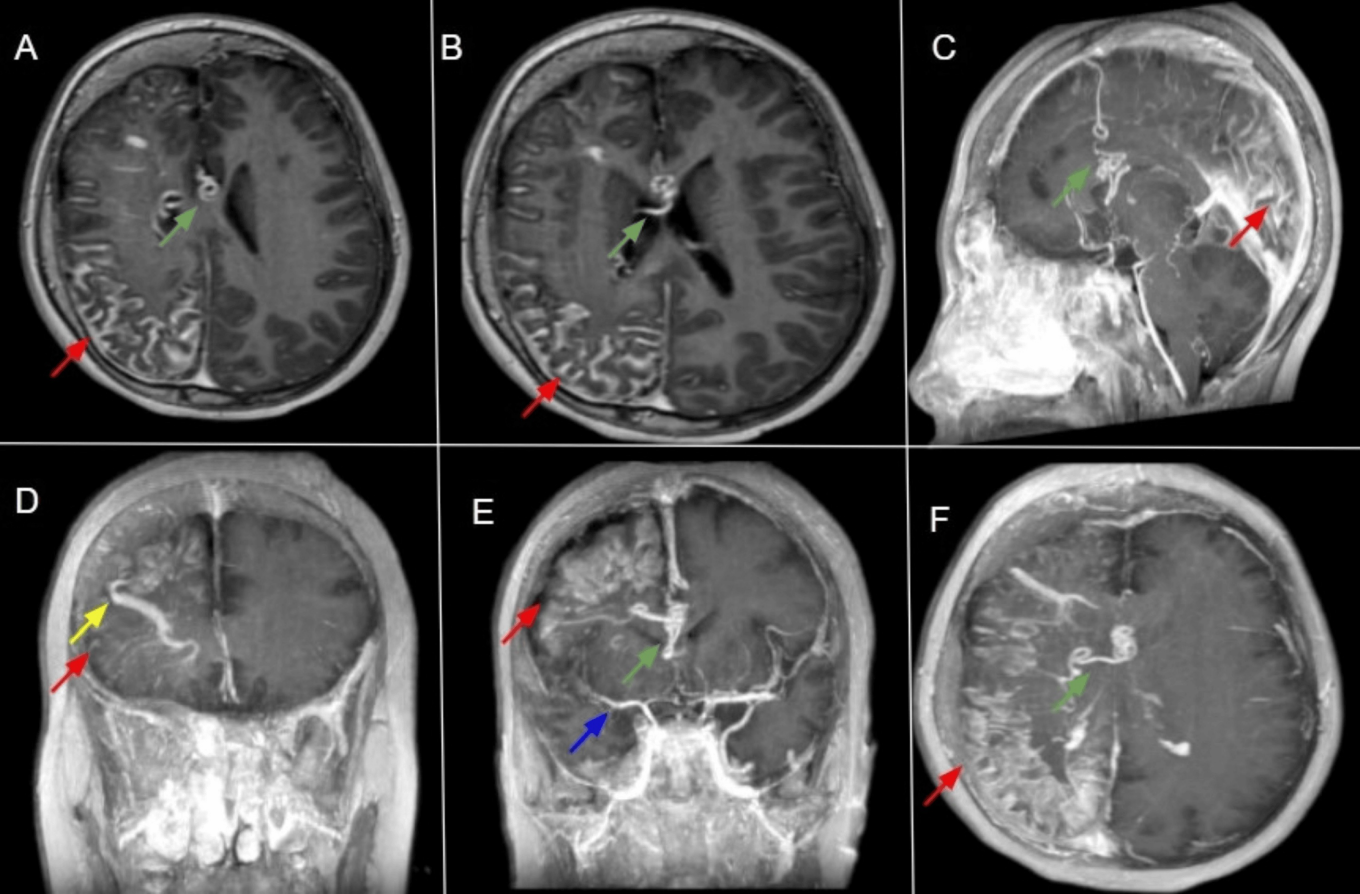
Axial post-contrast T1 non-fat-saturated images Axial post-contrast T1 non-fat-saturated images (A–B) and MIP reconstructions (C–F) demonstrate right cerebral pial angiomatosis (red arrows), a right-sided developmental venous anomaly (yellow arrow), and aberrant venous channels (green arrows) connecting prominent subependymal and superficial veins. Arterial abnormality is also seen, with a relative hypoplastic right middle cerebral artery (blue arrow). MIP: Maximum intensity projection.

Contrast-enhanced MRI sequences provided further delineation of the abnormal vascular architecture, demonstrating arterial narrowing, prominent pial angiomas, paucity of superficial cortical veins, and compensatory recruitment of deep subependymal venous drainage channels (hallmark features of this disease), as shown in Figure 8.

**Figure 9:**
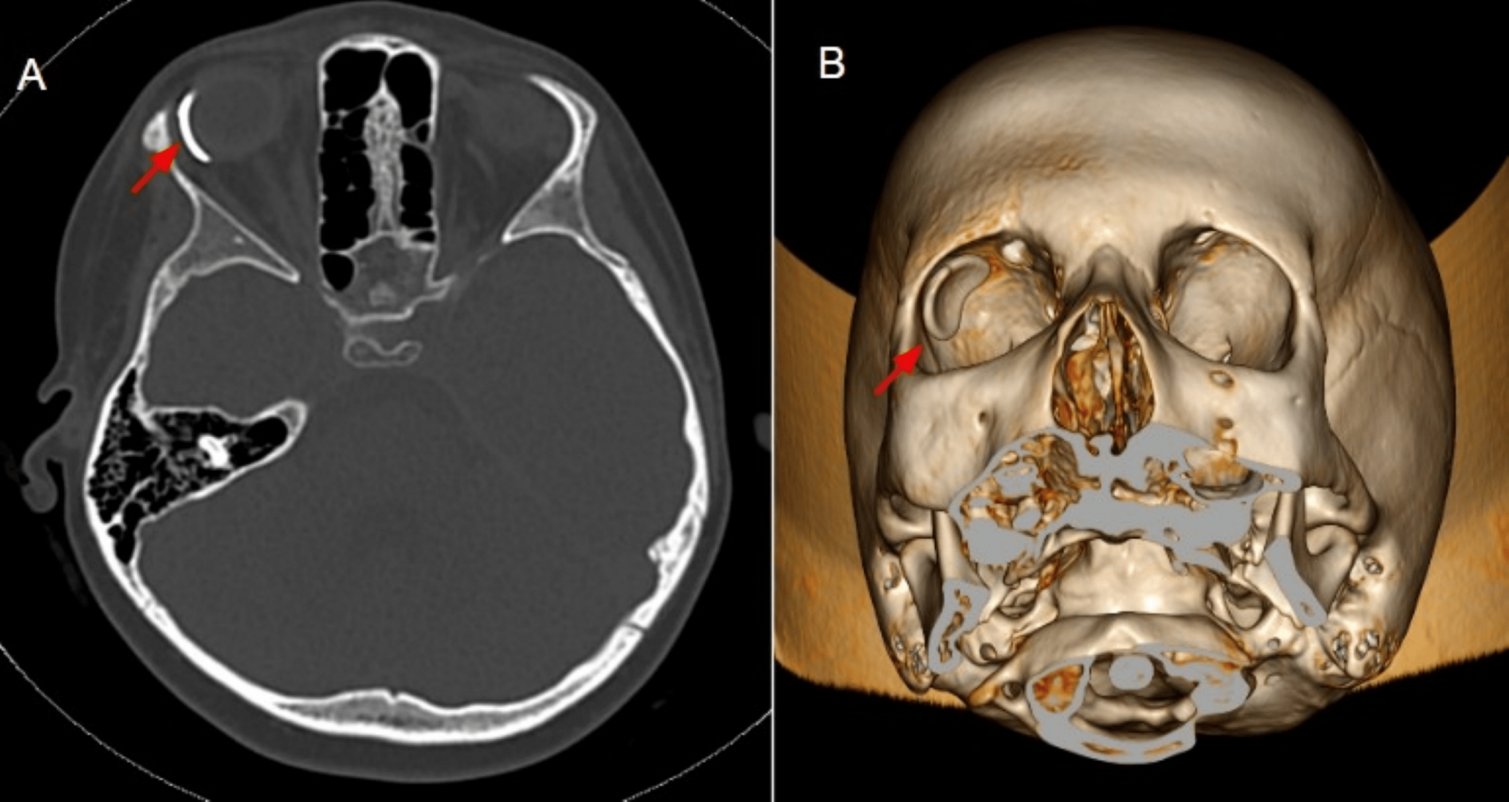
Axial CT bone window image Axial CT bone window image (A) and 3D reconstruction (B) demonstrate right eye Baerveldt tube implantation (red arrow) for glaucoma management, performed at seven years of age.

Clinically, seizure control has been achieved with carbamazepine and intermittent use of midazolam. Ocular involvement has been significant, with right-sided glaucoma initially managed using topical agents (latanoprost, timolol, and dorzolamide). At the age of seven years, a Baerveldt tube was implanted in the right eye to reduce intraocular pressure further, as shown in Figure 9.

Neurologically, she continues to experience seizures, although these are adequately controlled with medication. Currently, the patient is under multidisciplinary follow-up, including radiology. This longitudinal case study illustrates the progressive cerebrovascular pathology of SWS and underscores the importance of serial neuroimaging for monitoring disease progression. A summary of imaging findings across different ages is provided in Table 1.

**Table 1:** Longitudinal neuroimaging findings in a pediatric patient with Sturge–Weber syndrome NCCT: Non-contrast computed tomography; MRI: Magnetic resonance imaging, -: Absent/Not visualised. Severity grading: + minimal; ++ mild; +++ moderate; ++++ severe.

Patient’s age at the time of Imaging	One month	One Year	Six Years	10 Years	12 Years
Clinical concern	Facial port-wine stain	Fits	Prolonged seizure followed by left-sided weakness.	Developmental delay and seizure under control.	Routine follow-up scan
Imaging Modality	Contrast MRI Head	NCCT and Contrast MRI Head	NCCT and non-contrast MRI Head	NCCT and non-contrast MRI Head	Contrast MRI Head
Pial angiomatosis (Figure 2)	+	++	++	++	++
Other vascular anomalies (Figure 3)	-	+	+	+	+
Parenchymal calcification (Figure 4)	-	+	+++	++++	++++
Parenchyma Atrophy (Figure 5)	-	+	+++	++++	++++
Calveria hypertrophy (Figure 6)	-	-	++	+++	+++
Sinus enlargement (Figure 7)	-	-	+	++	+++
Choroid plexus enlargement	+	++	++	++	++
Right eye diffuse choroidal haemangioma.	+	+	+	+	+

## Discussion

Sturge-Weber Syndrome (SWS) is a neurocutaneous disorder presenting with multisystem involvement, most commonly affecting the skin, eyes, and brain. The hallmark dermatologic feature is a port-wine birthmark (PWB), typically involving the ophthalmic (V1) division of the trigeminal nerve; its absence makes SWS unlikely [3]. Larger or more extensive PWBs are associated with a higher risk of intracranial involvement [4]. In our patient, the presence of a facial PWB at birth corresponded with early neuroimaging findings of right cerebral pial angiomatosis, confirming the diagnosis in infancy.

Ocular manifestations frequently include glaucoma, which may present in infancy or later and requires lifelong monitoring [5]. The risk is most significant when both eyelids are affected [6]. In our patient, imaging demonstrated a diffuse right eye choroidal hemangioma from birth, underscoring the importance of early ophthalmologic monitoring and intervention. Neurological symptoms often begin with early-onset seizures, usually before one year of age, with severity correlating with long-term outcomes. Seizures arise from pial angiomatosis, cortical calcification, and cerebral atrophy, which also predispose to stroke-like episodes and neurological deficits [7,8]. The clinical course typically progresses through four phases: presymptomatic, progressive, stable, and late progressive [9]. In our patient, seizures began at one year of age, with serial imaging showing progressive right hemispheric calcifications and parenchymal atrophy correlating with the clinical course of left-sided weakness and developmental delay observed between five and 12 years.

Headaches and migraines are familiar sources of morbidity, sometimes more disabling than seizures [10]. Endocrine abnormalities, including growth hormone deficiency, central hypothyroidism, and partial hypopituitarism, occur more frequently in SWS and may impair growth and neurocognitive development [11]. Clinically, SWS is categorized into three types: Type I, characterized by facial and pial angiomas with or without glaucoma; Type II, involving facial angioma without intracranial disease but with possible glaucoma; and Type III, consisting of isolated pial angioma without facial lesions and typically no glaucoma [12]. 

Most cases occur sporadically and are associated with somatic activating mutations in the GNAQ gene on chromosome 9q21, which impair vascular development and result in the characteristic cutaneous and intracranial malformations [13]. The pathophysiology of Sturge-Weber Syndrome (SWS) is driven by pial angiomatosis, which creates a vascular "steal" phenomenon, resulting in reduced perfusion to the underlying cortex and white matter. This chronic hypoperfusion leads to localized cortical ischemia, progressive atrophy, and is usually confined to a single hemisphere.

Role of neuroimaging in SWS clinical care and monitoring

Neuroimaging is central to the diagnosis and management of Sturge-Weber Syndrome (SWS), aiding in the detection, monitoring of acute events, and guiding treatment decisions. Historically, plain skull radiographs were used to identify gyriform 'tram-Track 'calcifications, which typically appear between two and seven years, but this modality is now largely obsolete. Computed tomography (CT) remains more sensitive, enabling the earlier detection of subcortical calcifications, often accompanied by associated parenchymal volume loss. The extent of calcification correlates with seizure severity, earlier onset, and worse neurological outcomes [14]. Classic CT findings include tram-track calcifications, ipsilateral calvarial and sinus enlargement, enlarged choroid plexus, orbital choroidal haemangiomas, and asymmetric cavernous sinus enlargement. In severe cases, the appearance may mimic Dyke-Davidoff-Masson syndrome.

Magnetic resonance imaging (MRI) provides greater sensitivity for early changes. T1-weighted imaging may appear normal in infancy, with progressive cerebral volume loss evident over time. Post-contrast T1 with gadolinium typically demonstrates leptomeningeal enhancement corresponding to pial angiomatosis and venous congestion, which can progress to ischemia, infarction, and atrophy. Enhancement often decreases as the angioma regresses. Other findings include ipsilateral choroid plexus enlargement, dilated trans-parenchymal veins, and abnormal deep venous drainage. T2-weighted imaging may also reveal low signal in subjacent white matter, which was once attributed to accelerated myelination but is now recognized as early calcification. Gradient echo and susceptibility-weighted sequences are especially sensitive for detecting calcifications. MR spectroscopy often reveals reduced N-acetylaspartate (NAA), indicating neuronal loss [15].

Despite its important role, routine annual MRI is not recommended in clinically stable children due to the risks of sedation and contrast administration. Advanced MRI sequences like perfusion, etc., are usually indicated if any intervention/surgery is planned. Our patient demonstrated progressive gyriform calcifications on serial CT and MRI scans, with associated hemispheric atrophy, right hemicalvarial and paranasal sinus hypertrophy, and an enlarged choroid plexus. In our patient, we also observed a T2/FLAIR hyperintense signal in the subcortical white matter underlying pial angiomatosis (Figure 2c). These modifications most likely reflect hypoxic injury associated with long-term seizures, which leads to changes in the white matter and decreased cerebral perfusion [16]. 

Both the arterial and venous cerebral circulations exhibit an abnormal pattern in SWS. Venous changes are characterised by occlusions, scarcity of superficial draining veins, and alternate venous drainage through deep subependymal channels, whereas arterial changes include arterial thrombosis and narrowing [17]. Positron emission tomography (PET) further complements MRI: in young children, particularly before age two years, 18F-Fluorodeoxyglucose (18F-FDG) PET may reveal hypermetabolism in the affected hemisphere, often just before or around seizure onset. With disease progression, this pattern evolves into hemispheric hypometabolism, consistent with chronic neuronal loss and dysfunction [18].

The differential diagnosis of Sturge-Weber Syndrome (SWS) includes several rare conditions. Gobbi (CEC) syndrome, although uncommon, presents with bilateral occipital calcifications but does not exhibit hemispheric atrophy or leptomeningeal enhancement. Cerebral proliferative angiopathy is characterized by diffuse tubular or linear hyperdensities, multiple small feeding vessels and veins without a dominant nidus, and intervening normal brain tissue. Meningioangiomatosis can mimic SWS by presenting with focal cortical or subcortical nodular or gyriform lesions, calcifications, and enhancement; however, it typically spares white matter and does not cause hemispheric atrophy. Our patient demonstrated progressive gyriform calcifications on serial CT and MRI scans, with associated hemispheric atrophy, right hemicalvarial and paranasal sinus hypertrophy, and an enlarged choroid plexus. 

Management of Sturge-Weber Syndrome (SWS) is primarily symptomatic and supportive [19], with seizure control being the central therapeutic goal. First-line treatment involves anticonvulsants; however, their effectiveness may be limited, and a significant number of patients develop refractory seizures that necessitate surgical intervention [20]. Ophthalmologic management is crucial, as ocular involvement can lead to glaucoma and choroidal vascular malformations. Surgical interventions are particularly effective for glaucoma, whereas cosmetic treatment of port-wine stains is typically achieved through laser photothermolysis. Despite these measures, no definitive curative therapy exists for the underlying vascular malformations. Prognosis largely depends on the severity and extent of neurological and ocular involvement. Early diagnosis, combined with timely management, can significantly improve functional outcomes and quality of life.

## Conclusions

This longitudinal case of Sturge-Weber syndrome (SWS), followed from infancy through adolescence, demonstrates the predictable and progressive nature of its neuroimaging manifestations. Serial studies revealed the sequential evolution of hallmark features, including persistent pial angiomatosis, gyriform calcifications, progressive cerebral atrophy, ipsilateral calvarial hypertrophy, and choroid plexus enlargement, broadly paralleling the patient’s clinical course and seizure burden. These observations underscore the value of long-term neuroimaging in understanding the natural history of SWS and in correlating radiological progression with neurological outcomes.

Our experience reinforces the importance of early MRI in confirming the diagnosis, establishing a baseline for follow-up, and supporting multidisciplinary management. While treatment remains largely supportive, timely identification of complications such as refractory epilepsy and glaucoma can improve quality of life and functional outcomes. Importantly, imaging findings should be interpreted in conjunction with seizure control, neurodevelopmental progress, and ophthalmologic status. The frequency of neuroimaging should be individualized, guided by clinical changes and management needs, rather than performed routinely in stable children, to avoid unnecessary sedation or contrast exposure.
